# Conserved signatures indicate HIV-1 transmission is under strong selection and thus is not a “stochastic” process

**DOI:** 10.1186/s12977-016-0326-1

**Published:** 2017-02-24

**Authors:** Mileidy Gonzalez, Anthony L. DeVico, John L. Spouge

**Affiliations:** 1 Statistical Computational Biology Group, National Center for Biotechnology Information (NCBI), National Library of Medicine (NLM), National Institutes of Health (NIH), Bethesda, MD USA; 2 Division of Basic Science and Vaccine Research, Institute of Human Virology, University of Maryland School of Medicine, Baltimore, MD USA

**Keywords:** Transmitted/founder virus, T/F, Transmission signatures, Selection during HIV transmission, Phenotypic versus genotypic selection

## Abstract

Recently, Oberle et al. published a paper in *Retrovirology* evaluating the question of whether selection plays a role in HIV transmission. The Oberle study found no obvious genotypic or phenotypic differences between donors and recipients of epidemiologically linked pairs from the Swiss cohort. Thus, Oberle et al. characterized HIV-1 B transmission as largely “stochastic”, an imprecise and potentially misleading term. Here, we re-analyzed their data and placed them in the context of transmission data for over 20 other human and animal trials. The present study finds that the transmitted/founder (T/F) viruses from the Swiss cohort show the same non-random genetic signatures conserved in 118 HIV-1, 40 SHIV, and 12 SIV T/F viruses previously published by two independent groups. We provide alternative interpretations of the Swiss cohort data and conclude that the sequences of their donor viruses lacked variability at the specific sites where other studies were able to demonstrate genotypic selection. Oberle et al. observed no phenotypic selection in vitro, so the problem of determining the in vivo phenotypic mechanisms that cause genotypic selection in HIV remains open.

## Correspondence

Oberle et al. [1] recently analyzed nine linked HIV-1 subtype B transmission pairs from the Zurich Primary HIV Infection (ZPHI) study and the Swiss HIV Cohort Study (SHCS), generating a novel resource for information about HIV donor and recipient viruses. The authors used their data to address whether natural HIV transmission entails selection for particular phenotypic or genotypic characteristics, a question that remains critical to the successful development of preventive measures against HIV-1 infection. The authors concluded that their data favor a model in which HIV-1 transmission is largely “stochastic”. The terminology is in fact misleading, because the data are consistent with other evidence confirming that selection occurs during viral transmission. Our reasoning is described below.

### Phenotypic analyses in vitro may not detect genotypic selection in vivo

Oberle et al. examined viruses from donor–recipient pairs for certain phenotypic characteristics (e.g., neutralization and entry inhibitor sensitivity, replicative capacity, sensitivity to Interferon α, etc.). In their cohort, the phenotypic traits measured in vitro clearly displayed no selection, an interesting and useful finding. The breadth of their conclusions about the absence of selection in natural HIV transmission is less certain, however. The characteristics measured in vitro, although carefully chosen for probable importance in vivo, may have only indirect relevance to the natural transmissibility of HIV. Consider, e.g., neutralization sensitivity/resistance, which per se (depending on the route of exposure) may have little relevance to the rapid establishment of infection in a naïve, seronegative host. In summary, the experimental conclusions inevitably hold the caveat that in vitro assays may not be able to detect the in vivo selective pressures on viral genotypes.

### “Stochastic” is an imprecise term when applied to HIV transmission

A “stochastic process” is a random function. In the context of HIV transmission, it entails a probability model for random variables changing over time. The conclusion of “stochastic” transmission therefore only implies some degree of randomness, and it does not exclude biological selection. If a study concludes that transmission is “stochastic”, therefore, it should probably conclude more accurately (and certainly more clearly) that it did not detect selection. In fact, a precise statistical framework to test for selection asks the question: are T/F viruses chosen uniformly at random from the donor pool? The null hypothesis for statistical testing is therefore that T/F viruses are chosen uniformly at random from the donor pool.

### Absence of evidence is not evidence of absence

This popular aphorism summarizes our main objection with the conclusions of the Oberle study. Absence of evidence, or the failure to observe evidence supporting a hypothesis in a specific circumstance, is not necessarily broad evidence against the hypothesis. Thus, absence of selection (in a specific set of in vitro tests applied to a specific set of donor–recipient pairs) does not constitute evidence against selection and in favor of a uniform random process for all subtype B HIV-1 transmissions. Selection might even be playing a direct role in the transmissions in the Oberle study, but may not be evident perhaps because of unsuitable data, resolution, algorithms, tools, etc. The conclusions of the Oberle study therefore need to be appropriately qualified.

### Failure to reject the null hypothesis is not the same as accepting it

In science, it is standard to calculate p values. When a p value is significant, by convention, we reject the null hypothesis (e.g., H_0_ = T/F viruses are chosen uniformly at random from the donor pool). Insufficiently significant p values, as was the case for the comparisons in the Oberle study, are not enough to accept the null hypothesis. Data may simply be too sparse or inappropriate to detect weak trends supporting an alternative hypothesis (e.g., H_A_ = T/F viruses are under selection).

### Signatures in T/F viruses have already demonstrated selection during HIV transmission

**Fig. 1:**
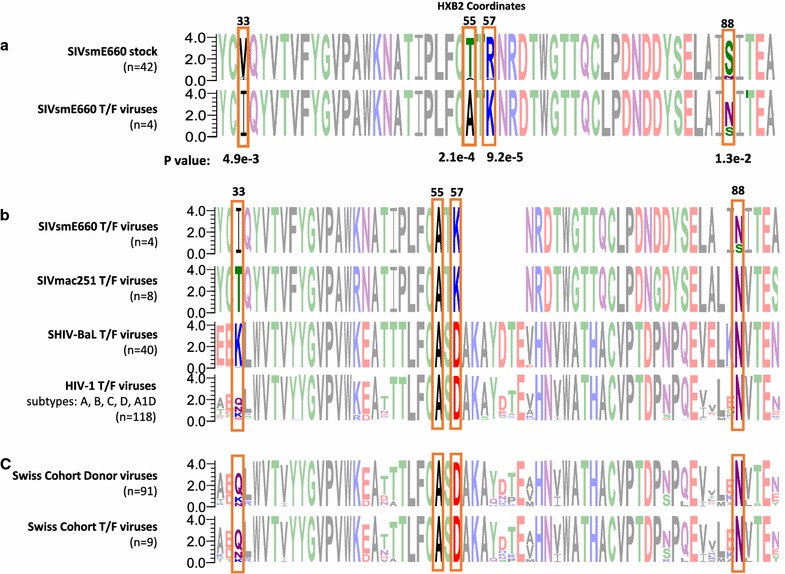
Transmission signatures conserved in SIV, SHIV, and HIV-1. **a** An alignment of two sequence logos representing Env sequences from an SIVsmE660 stock/inoculum (*top*) and the T/F (*bottom*) viruses establishing infections in four animals. The diagram illustrates the signature scanning protocol on one SIVsmE660 trial highlighting four signature sites: i.e., sites where the amino acid preference in the donor residue differs significantly from that of the T/F based on a Fisher Exact test. See our previous published work for statistical details of signature scanning in 11 other trials [3]. **b** Sequence logos tracking the signature residues in 170 T/F viruses from SIVsmE660, SIVmac251, SHIV-BaL, and HIV-1 (A, B, C, D, A1D). **c** An alignment of the donor (*top*) and T/F sequences from the study by Oberle et al. Sequence logos were generated using WebLogo software [16, 17]

The authors state: “To what extent HIV-1 transmission is a stochastic process or driven by selective forces that allow T/F viruses best to overcome bottlenecks in transmission has not been conclusively resolved.” In fact, the available literature on this subject consistently shows envelope sequence bias during transmission. Gnanakaran et al. [2] identified one signature in the Env signal peptide. We also identified four strong gp120 signatures conserved in T/F viruses from twelve different HIV-1, SHIV, and SIV trials [3]. Figure 1a illustrates our previous signature analysis on one of the SIV animal trials. The signature scanning algorithm identified that SIV T/F viruses were almost invariably Ile[33]-Ala[55]-Lys[57]-Asn[88] variants, despite I-A-K-N being a minor variant in the stock (2%). V-T-R-S constituted the major variant in the stock (79%); yet, none of the T/F viruses featured those residues at the signature sites (Fig. 1a). While other variants can sometimes transmit (e.g., IAKS), they do so infrequently and cannot compete in the presence of even miniscule proportions of I-A-K-N variants. Our findings of selection on T/F viruses were confirmed on data from four different vaccine trials by Smith et al. [4].

### The A-[DK]-N signatures are conserved in HIV-1, SHIV, and SIV T/F Viruses

The extreme diversity of the SIVsmE660 stocks made it possible to observe selection at the transmission signature sites (Fig. 1a). Our previous report demonstrated statistical support for the I-A-K-N signatures from three different trials challenging vaccinated and unvaccinated animals with SIVsmE660O [3–6]. T/F viruses from other strains show diversity at site 33; thus, the I33 transmission signature seems peculiar to SIVsmE660 (Fig. 1b). We also showed that the signatures at sites 55, 57, and 88 are conserved in T/F viruses from SIVmac251, SHIVBaL, and HIV-1 infections (Fig. 1b). The analogous signatures in HIV-1 and SHIV are A55-D57-N88. Note that we analyzed HIV-1 T/F viruses from subtype A [7], subtype B (the Step trial [8, 9], the Nairobi breastfeeding trial [10, 11], and Los Alamos database [2]), subtype C [12], subtype D [7], and subtype A1D [7] and SHIV T/F viruses from three different sources [3, 13]. For the sake of the present analysis, the signature algorithm scanned additional T/F viruses from one other SHIVBaL vaccine trial [14] and 13 infectious molecular T/F clones from the NIH AIDS reagents program. All T/F viruses we have collected in HIV-1, SHIV, and SIV show the A55-[DK]57-N88 signature regardless of sex, gender, vaccination status, viral dosage, or transmission route (Fig. 1b).

### The T/F viruses of the Swiss Cohort also display the same conserved transmission signatures reported previously in other HIV, SHIV, and SIV T/F viruses

In accordance with the above findings, the signature scanning algorithm illustrated in Fig. 1a applied to the genetic data in the Oberle et al. study reveals that the A55, D57, and N88 envelope residues are selectively conserved in the T/F viruses of the Swiss cohort (Fig. 1c). Unfortunately, the diversity and sparsity of donor sequences in the Swiss cohort (Fig. 1c) precludes direct statistical elucidation of these residues/positions as formal transmission signatures. It is worth mentioning that 3% of the Swiss donor sequences differ at site 57: i.e., 3% of the non-transmitted donor viruses are [EN]57 variants whereas all the Swiss T/F viruses are D57 variants. Still, since A55-D57-N88 viruses constitute the major variant in the Swiss donor and T/F viruses (Fig. 1c) there is not enough variation in the Swiss cohort alone to gather statistical evidence in support of selection. The same caveats make it impossible to draw the opposite conclusion; i.e., that there is no selective pressure at these sites during transmission (see above). However, using the observations of the 170 other T/F viruses (118 HIV-1, 40 SHIV, and 12 SIV T/F viruses in Fig. 1b) as a framework, it is entirely plausible to interpret A55, D57, and N88 as non-random markers of T/F viruses in the Swiss cohort.

## Conclusions

The Oberle et al. study found no evidence of phenotypic or genotypic selection in the transmitted viruses of the Swiss cohort; thus, the authors conclude that HIV transmission is largely “stochastic”. Here, we raise the issue that the breadth of their conclusions about the absence of selection in natural HIV transmission needs to be re-evaluated. On the one hand, it is well established that CCR5 tropism is a phenotypic determinant of HIV transmitted viruses indicating selection at the phenotypic level [15]. On the other hand, we and others have demonstrated selection at the genotypic level [3, 4].

It is clear that the viruses in the Swiss cohort alone cannot provide genotypic evidence of selection due to limited diversity of donor viral sequences. When considering the Swiss cohort sequences in the context of transmission data from over 20 other studies of human and non-human primate lentiviruses, we find that the Oberle et al. data conform to redundant findings that transmission is under strong selection to conserve the A55-[DK]57-N88 envelope residues.

Relatively limited diversity in donor sequences (in comparison to animal donor data) has been typical of every human trial thus far, which possibly results from limited sampling, shallow sequencing breadth, or from strong selection. The latter possibility is supported by findings that A55-[DK]57-N88 predominate also in database sequences of circulating variants from chronic infection [3, 4]. The A[DK]N motif seems to predominate in circulating HIV-1 and HIV-2 variants. Given the high mutation rates and viral turnover in HIV, such levels of conservation beyond transmission indicate strong selection for functional or structural fitness reasons.

Such observations indicate that the A55-[DK]57-N88 sites are “lethal”; specifically, introducing other residues therein extract an extreme fitness cost to the virus. What specific role the amino acid residues defining these putative markers have in increasing HIV transmission efficiency remains unclear. It should be noted that the genetic transmission signatures discussed above have yet to be directly linked with the phenotypic traits Oberle et al. analyzed in their cohort study. Accordingly, one interpretation of Oberle et al. is that the key phenotypic traits peculiar to HIV-1 T/F variants remain to be defined and could benefit from other advanced structural analyses, in situ imaging, and/or in vivo analyses of viral replication. Taken together, our evidence indicates that under phenotypic circumstances not examined in the Swiss cohort, HIV T/F viruses are undergoing selection.
